# Prevalence of soil-transmitted helminth infections and associated risk factors among elderly individuals living in rural areas of southern Thailand

**DOI:** 10.1186/s12889-020-09986-7

**Published:** 2020-12-07

**Authors:** Ratee Kache, Nonthapan Phasuk, Parnpen Viriyavejakul, Chuchard Punsawad

**Affiliations:** 1 School of Medicine, Walailak University, Nakhon Si Thammarat, Thailand; 2 Tropical Medicine Research Unit, Research Institute for Health Sciences, Walailak University, Nakhon Si Thammarat, 80160 Thailand; 3 Department of Tropical Pathology, Faculty of Tropical Medicine, Mahidol University, Bangkok, Thailand

**Keywords:** Soil-transmitted helminth infections, Tha Sala District, Prevalence, Risk factor, Elderly

## Abstract

**Background:**

Soil-transmitted helminth (STH) infection is a neglected tropical disease affecting approximately 1.5 billion people worldwide. In past decades, most studies focused on STH infection in preschool-aged and school-aged children in different regions of Thailand. However, little is known about the prevalence and intensity of STH infection in the elderly population. Therefore, the aim of this study was to determine the current prevalence and intensity of STH infections and to identify associated risk factors among the elderly population.

**Methods:**

A cross-sectional study was conducted from July to November 2019 to assess the prevalence of STH infections and associated risk factors among elderly populations living in five subdistricts of Thasala District, Nakhon Si Thammarat Province, Thailand. A total of 439 elderly individuals were selected using a random sampling technique. Each fresh stool sample was examined using the formalin ethyl acetate concentration technique (FECT), Kato-Katz thick smears and agar plate culture (APC). A structured questionnaire was used to obtain relevant information regarding associated risk factors for STH infection.

**Results:**

The overall prevalence of STH infection was 15.7%. Hookworms (10.9%, 48/439) were the most prevalent STH species, followed by *Strongyloides stercoralis* (3.4%, 15/439) and *Trichuris trichiura* (2.1%, 9/439). Most elderly individuals infected with hookworms or *T. trichiura* had light-intensity infections. A higher prevalence of STH infection was observed among individuals aged older than 80 years (23.4%) than among those aged between 70 and 79 years (15.2%) and 60–69 years (14.5%). Males were 1.85-times more likely to present with STH infections than females. Not washing vegetables before eating increased the risk of STH infection by 3.19 times, while defecation in an open field increased the risk of STH infection by 2.65 times.

**Conclusions:**

The findings suggested that STH infection is prevalent, and that hookworms are the most common STH species among elderly populations in southern Thailand. Personal hygiene and deworming programs should be implemented among the elderly population to reduce the risk and prevent the spread of STH infections.

## Background

Soil-transmitted helminth (STH) infections are considered a neglected tropical disease and include three main species of parasites: *Ascaris lumbricoides* (roundworm), *Trichuris trichiura* (whipworm) and *Ancylostoma duodenale* and *Necator americanus* (hookworms) [1–3]. The World Health Organization (WHO) estimated that more than 1.5 billion people are infected with STHs worldwide, and infection is widely distributed in tropical and subtropical areas [4]. Southeast Asia has been reported to have the highest prevalence of STH infection [5, 6]. Regarding age distributions and prevalence, ascariasis and trichuriasis mainly affect children, whereas hookworm affects both children and adults [1, 6]. Morbidity and mortality due to STH infections are related to the intensity of the infection in an affected person, as well as age and immunity [5]. In addition, several factors are related to the high prevalence of STH infections in tropical and subtropical countries, including climatic conditions, poor sanitation, a lack of safe water and poor hygiene practices [1, 7].

In Thailand, intestinal parasitic infections are prevalent in all regions; however, STH infections, especially hookworm infections, are particularly prevalent in the southern region of Thailand [8–11]. The prevalence of hookworm infection in both the Philippines and Thailand increases in the 20–29-year-old age group and remains high across all older age groups [6]. The prevalence rates of parasitic infections among children in neighboring countries were as high as 69.7% in Myanmar [12] and 98.4% in Malaysia [13]. In southern Thailand, hookworms are most common (15.8%), followed by *T. trichiura* (3.9%) and *A. lumbricoides* (1.7%), and the highest infection intensities are found in adults [8]. Recently, a prevalence survey of intestinal parasite infections conducted by village health volunteers in rural communities in southern Thailand revealed that people aged older than 40 years had a higher prevalence of helminth infection than those aged younger than 40 years [10], which was consistent with the result of a systematic review focusing on STH infection in Southeast Asia; the review demonstrated a high prevalence of hookworm infection in older age groups [6]. However, no studies have focused on STH infection in the elderly population in southern Thailand.

The number of older people in Thailand has increased since 1990 and is expected to increase through 2040, and population growth estimates range from 696.6 to 769.8% in 2020 and 2040, respectively [14]. Previous studies indicated that elderly individuals were infected with intestinal parasites at rates of 16.2 and 17.7% in Surin Province, northeastern Thailand and Chachoengsao Province, central Thailand, respectively [15, 16]. Furthermore, the rate of hookworm infections was 5.7% in Songkhla Province, southern Thailand [17]. Currently, Nakhon Si Thammarat, a province in southern Thailand, has an aging society. Considering the rapid increase in the aging population, this study aimed to investigate the prevalence and risk factors of STH infections in elderly people in Tha Sala District, Nakhon Si Thammarat Province, Thailand. Knowledge of the distribution and extent of STH infections is a prerequisite for planning and evaluating intervention programs.

## Methods

### Study design and setting

This cross-sectional study was conducted over a four-month period from July to November 2019 in Tha Sala District, Nakhon Si Thammarat Province, Thailand. Tha Sala District is located at 8°40′0″N latitude and 99°55′54″E longitude and has a total area of 363.891 km^2^; it is approximately 750 km from Bangkok, the capital city of Thailand. The average temperature in southern Thailand is 27.7 °C, with a minimum of 27 °C in January and a maximum of 28.5 °C in May. The annual rainfall is 1959.2 mm (Climatological Center, Thai Meteorological Department, Annual Report 2018). Tha Sala District is divided into 10 subdistricts, which are further subdivided into 110 administrative villages, and the total population was 113,323 in 2019 (The Official Statistics Registration System, Thailand Department of Provincial Administration). Most land is used for farming, followed by rubber plantations and fishing.

### Study population and sample size

**Fig. 1:**
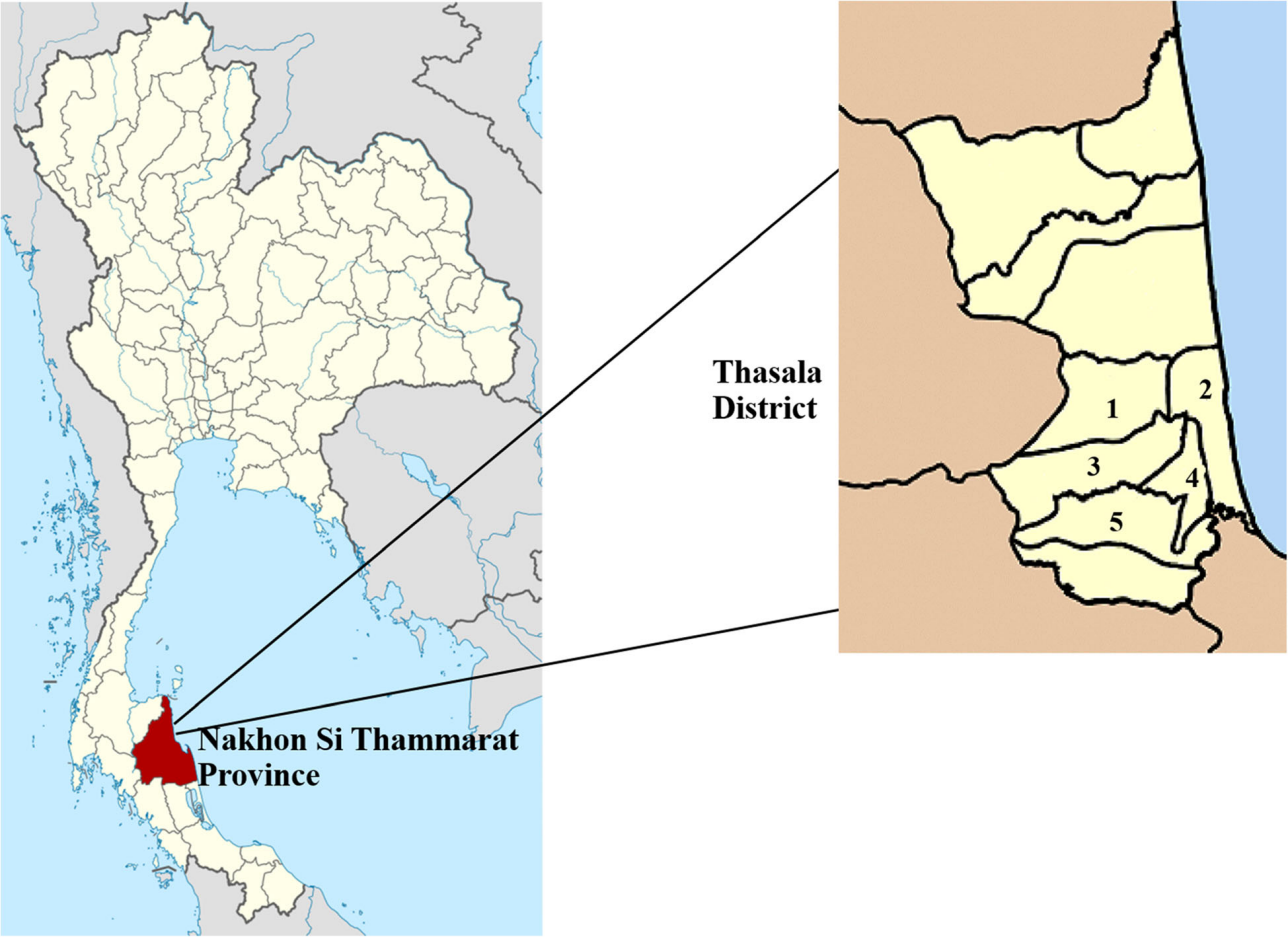
Map of Tha Sala District, Nakhon Si Thammarat Province, southern Thailand. Thai Buri (1), Tha Sala (2), Hua Taphan (3), Pho Thong (4) and Mokkhalan (5). (Map from https://commons.m.wikimedia.org/wiki/Atlas_of_Thailand)

The target study population was elderly people living in five subdistricts of Thasala District: Thai Buri, Tha Sala, Hua Tapan, Mokhalan, and Pho Thong (Fig. 1). The number of elderly individuals (aged 60 years and older) at the time was 16,968. The sample size was determined by the statistical formula *n* = N/1 + Ne^2^ [18], where N is the size of the elderly population in Tha Sala District, and e is the error tolerance level of 0.05 at the 95% confidence level. The calculated sample size was 391. To minimize errors caused by exclusion due to incomplete data or inadequate stool samples, the sample size was increased by 10%. The final sample size of this study was 430 individuals. The actual sample numbers of elderly participants from each subdistrict were determined by a random sampling technique.

### Questionnaire survey

A structured questionnaire was prepared to gather relevant information from each participant. Two domains were addressed in the questionnaire: sociodemographic (i.e., age, sex, monthly family income and education level) and risk factors for STH infections (i.e., handwashing before meals, eating fresh vegetables and wearing shoes). The content validity of the questionnaire was assessed by three experts in the field of parasitology or related fields. The reliability of the questionnaire was pretested with thirty individuals in a nonstudy sample population who lived outside the study area, and Cronbach’s alpha coefficient for the questionnaire was calculated to be 0.827.

### Stool sample collection and parasitological examination

All participants were given a stool container labeled with an identification number and asked to submit a fresh stool sample of approximately 10 g the next day. Each stool sample was assessed for correct labeling and an adequate sample quantity and transferred immediately to the parasitology laboratory of Walailak University. For parasitological analysis, each stool sample was examined for STH parasites by the formalin ethyl acetate concentration technique (FECT) to detect the eggs and larvae of helminths and Kato-Katz thick smears to quantify the number of helminth eggs excreted in stool (expressed as eggs per gram, EPG) following WHO and Centers for Disease Control standard operating procedures [19]. The intensity of STH infection was classified into three categories, namely, light, moderate or heavy intensity, according to WHO recommendations [20]. Additionally, the agar plate culture (APC) technique [21] was used to detect the presence of *S. stercoralis* in each specimen. In brief, two grams of fresh stool sample was placed on the center of nutrient agar plates and incubated for 3–5 days at room temperature [21]. The surface of the agar plate was examined under a stereomicroscope on the third or fifth day for the presence of either rhabditiform or filariform larvae. Each sample was prepared and examined in duplicate. All parasitological examinations were performed by an experienced medical technologist who was blinded to the information of the participants. For examination quality control, 10% of the stool samples were re-examined by another experienced parasitologist.

### Data analysis

The data were entered into an Excel database and double-checked to validate all data before analytical processing. All data analyses were performed using IBM SPSS Statistics for Windows, Version 23.0 (SPSS, Chicago, IL, USA). The elderly individuals were classified into 3 age groups: 60–70 years, 70–80 years and older than 80 years. Means and standard deviations (SD) were used to describe the quantitative factors, while frequency (%) was used to describe the qualitative factors. Proportions with 95% confidence intervals (CIs) were used to describe the prevalence of intestinal parasites. Initially, the chi-square test was used to assess differences in the presence of intestinal parasites by age group and subdistrict. Univariable analysis was utilized to examine the crude odds ratio (COR) of the binary outcome variable for each independent variable. All variables with *P*-values less than 0.2 in the univariable analysis were subjected to multivariable analysis to adjust for possible confounders. The final analysis was interpreted as adjusted odds ratios (AORs) with 95% CIs. *P*-values less than 0.05 were considered statistically significant.

## Results

### Sociodemographic characteristics

A total of 439 elderly individuals comprising 295 females (67.2%) and 144 males participated in the study. The mean age of the participants was 69.7 (standard deviation 7.2) years. Most (241/439) of the elderly individuals were between the ages of 60 and 70 years. More than half (74.26%) of the participants were Buddhists. Almost all (91.8%) of the study participants had an elementary-level education. Most of the participants’ occupations were agriculturists. The average monthly household income of most of the participants was less than 10,000–20,000 baht (Table 1).

**Table 1:** Sociodemographic characteristics of the elderly participants in Tha Sala District, Nakhon Si Thammarat Province, southern Thailand (*n* = 439)

Characteristics		Number	Percentage
Sex	Male	144	32.80
	Female	295	67.20
Age group	60–69 years	241	54.90
	70–79 years	151	34.40
	> 80 years	47	10.71
Subdistrict	Tha Sala	90	20.50
	Hua Taphan	89	20.27
	Moklan	85	19.36
	Pho Thong	87	19.82
	Thai Buri	88	20.05
Religion	Buddhism	326	74.26
	Islam	113	25.74
Marital status	Single	25	5.69
	Married/Couple	289	65.83
	Widowed/Divorced	125	28.47
Education	Elementary education	403	91.80
	Secondary education	17	3.87
	Higher education	19	4.43
Occupation	Agriculturist	187	42.60
	Hireling	46	10.48
	Retired government official	8	1.82
	Merchant/Private business worker	29	6.61
	Housekeeper/Housewife/Househusband	83	18.91
	Unemployed	86	19.59
Monthly household	Less than 10,000 baht	256	58.31
Income (Thai baht)	10,001-20,000	140	31.89
	20,001-30,000	33	7.52
	Greater than 30,001	10	2.28

### Prevalence of STH infections

Of the 439 individuals who were examined for STH infection, the overall prevalence of STH infections among elderly participants was 15.7% (69/439). Most of the positive individuals had a single infection (92.8%, 64/69), whereas five participants (7.2%, 5/69) had multiple infections, including five cases of hookworm and *T. trichiura* coinfection and only one case of hookworm and *Enterobius vermicularis* coinfection. The most prevalent STHs were hookworms at 10.9% (48/439), followed by *Strongyloides stercoralis* at 3.4% (15/439) and *T. trichiura* at 2.1% (9/439) **(**Table 2**).** *A. lumbricoides* was not detected in any of the specimens examined. In addition, *E. vermicularis* was found in one of 439 participants. A higher prevalence of STH infection was observed in the elderly population aged more than 80 years (23.4%) than in the populations aged 70–79 years (15.2%) and 60–69 years (14.5%). According to the sex of the participants, male participants had an overall prevalence of 20.8% (30/114), whereas the infection rate among female participants was 13.2% (39/256), and a significant difference in infection rates was found between male and female participants (*X*^*2*^ = 4.23, *P* = 0.04) **(**Table 2**).**

**Table 2:** Prevalence of soil-transmitted helminth (STH) infections stratified by sex, age group and subdistrict

		STH species			
		Hookworms n (%)	*Strongyloides stercoralis* n (%)	*Trichuris trichiura* n (%)	Total n (%)
Sex	Male (*n* = 144)	20 (13.9)	8 (5.6)	3 (2.1)	30 (20.8)
	Female (*n* = 295)	28 (9.5)	7 (2.4)	6 (2.0)	39 (13.2)
	Overall (*n* = 439)	48 (10.9)	15 (3.4)	9 (2.1)	69 (15.7)
	*X*^*2*^	1.921	2.97	0.001	4.234
	*P*-value	0.166	0.085	0.973	0.040*
Age group (years)	60–69 (*n* = 241)	22 (9.1)	9 (3.7)	4 (1.7)	35 (14.5)
	70–79 (*n* = 151)	17 (11.3)	5 (3.3)	3 (2.0)	23 (15.2)
	> 80 (*n* = 47)	9 (19.1)	1 (2.1)	2 (4.3)	11 (23.4)
	*X*^*2*^	4.08	0.315	1.324	2.383
	*P*-value	0.130	0.854	0.516	0.304
Study site	Tha Sala (*n* = 90)	2 (2.2)	2 (2.2)	0 (0.0)	4 (4.4)
	Hua Taphan (*n* = 89)	8 (9.0)	3 (3.4)	2 (2.2)	12 (13.5)
	Moklan (*n* = 85)	16 (18.8)	5 (5.9)	4 (4.7)	24 (28.2)
	Pho Thong (*n* = 87)	11 (12.6)	2 (2.3)	2 (2.3)	14 (16.1)
	Thai Buri (*n* = 88)	11 (12.5)	3 (3.4)	1 (1.1)	15 (17.0)
	*X*^*2*^	13.275	2.285	5.279	19.15
	*P*-value	0.010*	0.684	0.260	0.399

*Statistically significant at *P* < 0.05

Regarding the intensity of STH infection, the mean numbers of EPG in stool were 143.88 EPG (SD: 339.39) and 63.95 EPG (SD: 46.44) for hookworm and *T. trichiura*, respectively. Of the 48 elderly individuals who were positive for hookworm infection, most had light-intensity hookworm infections (97.9%, 47/48), and only one had a moderate-intensity infection. All elderly individuals infected with *T. trichiura* had low-intensity infections.

The prevalence of STH infections ranged from 4.4 to 28.2% according to study sites. The highest prevalence of STH infections was observed in the Moklan subdistrict (28.2%), followed by the Thai Buri (17.0%), Pho Thong (16.1%), Hua Taphan (13.5%) and Tha Sala (4.4%) subdistricts. A significant difference in the infection rate was observed among the study sites (*X*^*2*^ = 19.15, *P* = 0.001) **(**Table 2**).**

### Potential risk factors for STH infection in elderly individuals

The results of the univariable and multivariable analyses of the risk factors associated with STH infections are shown in Table 3. Univariable analysis revealed that males were 1.72-times more likely to have STH infections than females (AOR = 1.72; 95% CI 1.02–2.92). A significant association was also identified between STH infections and washing vegetables before eating (COR 3.16; 95% CI: 1.73–5.76). No significant associations were found between STH infection and age group, occupation or monthly family income (*P* > 0.05) (Table 3). The multivariable analysis also showed that sex, washing vegetables before eating and defecation in a pit toilet or on the soil in a garden were significantly associated with STH infections. Males were 1.85 times more likely to develop STH infections than females (AOR = 1.85; 95% CI 1.07–3.19). Elderly people who did not regularly practice vegetable washing were 3.19 times more likely to have STH infections than those who washed vegetables before eating them (AOR = 3.19; 95% CI 1.70–6.00). Defecation in an open field increased the risk of STH infection by 2.65 times (AOR = 2.65; 95% CI 1.14–6.15) (Table 3).

**Table 3:** Univariable and multivariable analyses of risk factors associated with soil-transmitted helminth (STH) infections in the participants

Variables	Positive No. (%)	Negative No. (%)	Total *n* = 439	COR (95% CI)	*P*-value	AOR (95% CI)	*P-*value
Sex							
Male	30 (20.8)	114 (79.2)	144 (32.8)	1.72 (1.02–2.92)	0.041*	1.85 (1.07–3.19)	0.027*
Female	9 (13.2)	56 (86.8)	295 (67.2)				
Age in years							
60–69	35 (14.5)	206 (85.5)	241 (54.9)				
70–79	23 (15.2)	128 (84.8)	151 (34.4)	0.56 (0.26–1.19)	0.132	1.05 (0.58–1.90)	0.881
> 80	11 (23.4)	36 (76.6)	47 (10.7)	0.59 (0.26–1.32)	0.198	2.01 (0.91–4.46)	0.885
Monthly family income (Thai baht)							
< 10,000	35 (13.7)	155 (82.9)	256 (57.3)	0.69 (0.41–1.16)	0.165	0.83 (0.05–1.44)	0.511
≥ 10,001	34 (18.6)	215 (85.3)	183 (41.7)				
Occupation							
Agriculturist	32 (17.1)	155 (82.9)	187 (42.6)	1.20 (0.72–2.01)	0.490		
Nonagriculturist	37 (14.7)	215 (85.3)	252 (57.4)				
Wearing shoes when outside							
No	24 (17.8)	111 (82.2)	135 (30.2)	1.24 (0.72–2.14)	0.430		
Yes	45 (14.8)	259 (85.2)	304 (69.2)				
Wearing boots when tapping rubber/gardening							
No	27 (16.1)	141 (83.9)	168 (38.3)	0.96 (0.57–1.62)	0.873		
Yes	42 (15.5)	229 (84.5)	271 (61.7)				
Fresh vegetable consumption							
No	31 (16.9)	152 (83.1)	183 (41.7)				
Yes	38 (14.8)	218 (85.2)	256 (58.3)	0.86 (0.51–1.43)	0.552		
Washing vegetables before eating							
No	21 (31.8)	45 (68.2)	66 (15)	3.16 (1.73–5.76)	< 0.001*	3.19 (1.70–6.00)	< 0.001*
Yes	48 (12.9)	325 (87.1)	373 (85)				
Handwashing before eating							
No	11 (16.2)	57 (83.8)	68 (15.5)	1.01 (0.52–2.11)	0.910		
Yes	58 (15.6)	313 (84.4)	371 (84.5)				
Handwashing after defecation							
No	4 (26.7)	11 (73.3)	15 (3.4)	2.01 (0.62–6.50)	0.245		
Yes	65 (15.3)	359 (84.7)	424 (96.6)				
Handwashing after contact with soil							
No	6 (15.4)	33 (84.6)	39 (8.9)	0.97 (0.39–2.42)	0.952		
Yes	63 (15.8)	337 (84.3)	400 (91.1)				
Defecating in an open field							
No	60 (14.8)	346 (85.2)	406 (92.7)	2.16 (0.96–4.88)	0.063	2.65 (1.14–6.15)	0.023*
Yes	9 (27.3)	24 (72.7)	33 (7.5)				

*Statistically significant at *P* < 0.05

## Discussion

This study was conducted in Nakhon Si Thammarat Province, an area endemic for soil-transmitted helminthiasis according to past and present studies and reports [8–10]. In our study, the overall prevalence of STH infections in the elderly population was 15.7%, which is consistent with the rate of intestinal parasitic infections among elderly individuals (17.7%) in a previous study conducted in Chachoengsao Province [15]. The prevalence in this study was lower than those reported in other studies conducted in different regions of Thailand, including Chanthaburi Province (29.6%) and Surin Province (16.2%) [16]. In contrast, the overall prevalence in this study was higher than those in previous studies in Nakhon Si Thammarat Province (9.3%) and Songkhla Province (5.3%) [10, 17]. The observed discrepancy might be due to differences in the climate, geographic location, age of the study population, sampling techniques, sociodemographic features and culture.

Hookworms were the most predominant STH parasites, consistent with a previous study from China demonstrating that hookworms were the most prevalent in the elderly population [22]. In addition, the observed hookworm infection prevalence of 10.9% was higher than those in rural communities of Chachoengsao Province [15], Khon Kaen Province [23], Surin Province [16] and Songkhla Province [17]. In our study, the prevalence of hookworm infection in elderly individuals aged more than 80 years (19%) was higher than those in individuals aged 60–69 years (9.1%) and 70–79 years (11.3.4%). The second most predominant parasite was *S. stercoralis,* with a prevalence rate of 3.4%, according to APC. Regarding the parasitological techniques for the diagnosis of *S. stercoralis*, APC has been recommended for epidemiologic purposes when compared with the FECT [24]. The prevalence of *S. stercoralis* in the elderly population was higher than that in a previous study conducted among adult populations in Nakhon Si Thammarat Province (1.0%) using APC [10]. In addition, the lowest prevalence of STHs was 2.1% for *T. trichiura,* which is higher than those in previous reports conducted in central [15] and southern Thailand [10]. No *A. lumbricoides* was detected in this study, which was consistent with a study of schoolchildren and adults conducted in Nakhon Si Thammarat Province [8–10]. In contrast, *A. lumbricoides* was commonly detected in studies conducted in northern Thailand and northeastern Thailand [8, 25–27]. Differences in the prevalence may be due to several factors, including variations in examination techniques, geographical areas, age groups, levels of education, occupations, food consumption habits, and personal hygiene behaviors [8, 16, 17, 28].

In this study, almost all the people with STH infections had light-intensity infections. Only one case of moderate-intensity hookworm infection was noted. These findings are consistent with previous studies in southern Thailand [8, 10].

Our study demonstrated a significant association between male sex and STH infection. Males had a higher prevalence of STH infections than females, consistent with previous studies in Thailand [10, 29]. We found that males were 1.85 times more likely to be infected with STHs than females. The high prevalence in males might be because males engage in outdoor activities more, making them more susceptible to STH infections than females. In the comparison of all age categories, this study showed that elderly individuals aged more than 80 years had a slightly higher prevalence of STH infections than those younger than 80 years. This result was in contrast to another study conducted among elderly individuals in Brazil that revealed that elderly individuals aged more than 80 years had a lower prevalence of STH infections than younger people [30]. However, a limited number of participants were aged older than 80 years in that study; thus, the result might not truly represent the rate of STH infections among the extremely elderly population.

Regarding behavioral risk factors, logistic regression analyses revealed that consuming unwashed fresh vegetables and defecation in an open field were significantly associated with STH infection. Elderly individuals who did not regularly practice vegetable washing were 3.19 times more likely to be infected with STHs than those who did wash vegetables. Several studies have reported that consuming unwashed fresh vegetables is a risk factor for STH infection [31, 32]. Recently, contamination by STH parasites has been reported in fresh vegetables from southern Thailand, with a contamination rate of 35.1% [32]. Therefore, the participants who lived in this area might have possibly acquired STH infections from ingesting STH-contaminated fresh vegetables [32, 33]. This study found that elderly individuals who defecated in an open field had a 2.65 times increased risk of STH infection. Unhygienic defecation habits are associated with a high risk of STH infection, especially infection by *S. stercoralis,* and hookworms that live in soil can be directly transmitted from the soil to humans through skin penetration by filariform larvae (the infectious stage) [34, 35]. Moreover, open defecation can cause dispersion of STHs as well [36]. This study found that other factors such as age, educational level, monthly household income, consumption of partially-cooked food, handwashing before eating and occupation were not significantly associated with STH infection, consistent with previous studies in Thailand [10, 15].

The results of this study confirmed that STH infections are not only limited to children and adults in the study area [9, 10] but are also prevalent among elderly individuals. We suggest that health education and deworming programs should also be implemented among elderly individuals who may undermine the progress of control activities by serving as sources of infections to children and other family members.

The study limitations are as follows. In this study, we obtained only a single stool specimen from each participant, which might underestimate the presence of parasites, as the standard specimen collection protocol is three specimens with intervals of 3 days. Another limitation of this study was the lack of information on clinical symptoms. We did not gather data on underlying medical conditions or the immune status of the participants, which might be possible confounders for STH infection. Furthermore, this study was limited to only elderly individuals in a region of southern Thailand. Additional studies should be performed in urban or rural areas and in different parts of the country.

## Conclusions

The findings suggested that STH infection is prevalent, and that hookworms are the most common STH species among elderly populations in southern Thailand. Male sex, consumption of unwashed fresh vegetables, and defecation in an open field increased the risk of STH infections. Health education and deworming programs should be implemented among the elderly population to improve health as well as household sanitation.

## Data Availability

All data generated or analyzed during this study are included within the published article.

## Abbreviations

STH: Soil-transmitted helminth
EPG: Eggs per gram
AOR: Adjusted odds ratio
CI: Confidence interval
IQR: Interquartile range
COR: Crude odds ratio
WHO: World Health Organization
